# Microcirculatory parameters as risk factors for predicting progression of posterior staphyloma in highly myopic eyes: a case–control study

**DOI:** 10.1186/s40662-024-00413-1

**Published:** 2024-12-01

**Authors:** Haoru Li, Nan Gao, Ruixin Li, Luobu Luodian, Jinyuan Sui, Yang Bai, Di Wu, Qing He, Yuxin Wang, Zhiqing Li, Ruihua Wei

**Affiliations:** Tianjin Key Laboratory of Retinal Functions and Diseases, Tianjin Branch of National Clinical Research Center for Ocular Disease, Eye Institute and School of Optometry, Tianjin Medical University Eye Hospital, Tianjin, China

**Keywords:** High myopia, Posterior staphyloma, Macular curvature, Microcirculation, OCTA

## Abstract

**Background:**

To assess the rate of macular blood flow decreasing in adults with and without posterior staphyloma (PS) using optical coherence tomography angiography (OCTA) and to identify risk factors associated with PS progression.

**Methods:**

This longitudinal case-control study enrolled 122 eyes of 122 patients—64 patients with PS (PS group) and 58 patients without PS (NPS group). Participants underwent OCTA and clinical examinations at least twice, and those followed for at least one year were included in the analysis. Logistic regression analysis and machine learning were applied to explore the risk factors for PS and its progression.

**Results:**

Patients in the PS group exhibited faster growth rates of spherical equivalent refraction (SER), axial length (AL), curvature index (CI), and posterior scleral height (PSH) as well as higher loss rates of choriocapillaris perfusion area (CCPA), choroid perfusion area (CPA) and choroidal vascularity index (CVI) compared to the NPS group (all *P* < 0.05). The baseline SER (B =  − 1.291, OR = 0.275, *P* = 0.008), baseline subfoveal scleral thickness (B =  − 1.621, OR = 0.198, *P* = 0.046), baseline PSH (B = 2.959, OR = 19.282, *P* = 0.001) and foveal CVI changes per year (B =  − 2.776, OR = 0.062, *P* < 0.001) were the risk factors for PS. Baseline AL (B = 0.561, OR = 1.752, *P* = 0.033), parafoveal choroidal thickness changes per year (B =  − 0.094, OR = 0.910, *P* = 0.032), foveal retinal vascular density changes per year (B = 0.104, OR = 1.110, *P* = 0.013) and foveal CCPA changes per year (B =  − 0.214, OR = 0.807, *P* = 0.038) were the risk factors for the PS progression.

**Conclusions:**

During the progression of myopia in adults, changes in the morphology of the eye's posterior pole are not limited to axial lengthening alone; there also will be a phase of compensatory lateral expansion. Baseline AL and changes in the microcirculation can be utilized to predict the progression of PS.

**Supplementary Information:**

The online version contains supplementary material available at 10.1186/s40662-024-00413-1.

## Background

The increasing prevalence of myopia has become a major public health problem worldwide. After the next 30 years, almost 1 billion people are expected to have high myopia [1]. High myopia is usually accompanied by myopic complications that can seriously damage visual acuity and cannot be prevented or treated by optical correction [2]. Posterior staphyloma (PS) has been considered as localized bulging of the eyewall, which is a typical sign of pathologic myopia [3]. The prevalence of PS has been reported to range from 10.9% to 50.5% [3–5]. The reasons for such large variation in the prevalence of PS, besides the differences in the study populations, the differences in the factors that affect PS have yet to be explored. The sclera, choroid, and Bruch’s membrane are considered to be associated with the pathogenesis of PS [6]. Abnormalities in macular microcirculation have been confirmed in several studies as an associated factor in high myopia and its complications [7–10]. The results of our preliminary cross-sectional study showed that changes in macular microcirculation are strongly associated with the existence of PS [11]. However, only a few studies have observed longitudinal changes in microcirculation in highly myopic eyes. Current research has focused on preventing the onset of myopia, but few studies have investigated how to slow the progression of myopic complications. If biomarkers can be found to predict myopic complications, it will be possible to intervene early and prevent further vision loss. With the development of optical coherence tomography (OCT) and optical coherence tomography angiography (OCTA) technologies, blood circulation in the fundus has become a quantifiable parameter [12], which provides a factor for exploring the pathogenesis of PS.

Since our study focused on the macula, the types of PS we included were mainly types I and II [13]. The purpose of this study was to observe the characteristics of macular curvature and macular microcirculation over time in highly myopic eyes with PS, aiming to investigate the characteristics of the progression and risk factors of PS.

## Methods

### Study participants

This is an observational longitudinal with case-control study. All study procedures adhered to the tenets of the Declaration of Helsinki and were approved by the ethics committee of the Tianjin Medical University Eye Hospital [No. 2021KY(L)-12]. Written informed consent from all participants were obtained. A total of 122 eyes of 122 patients were recruited from Tianjin Medical University Eye Hospital. All patients completed at least two follow-up visits. The initial inclusion criteria were age > 18 years; intraocular pressure (IOP) between 10 and 21 mmHg (1 mmHg = 0.133 kPa); spherical equivalent refraction (SER) ≤  − 6.00 D, and AL ≥ 26.0 mm; follow-up time longer than 12 months. Exclusion criteria were patients with systemic or ocular diseases, such as glaucoma, diabetes or hypertension; a history of myopia treatment, such as pharmaceutical interventions or ocular surgeries; eyes with retinal detachment, macular hole and choroidal neovascularization; poor quality OCT/OCTA images, which affected the automatic analysis of the machine.

### Ophthalmic examinations

All patients underwent a complete ophthalmic examination, including slit-lamp biomicroscopy, best-corrected visual acuity (BCVA), IOP (CT-1; Topcon, Japan), free mydriatic fundus photography (CR-2, Canon, Japan), axial length (AL) (Lenstar LS-900, Haag-Streit AG, Switzerland), SER (KR-800, Topcon, Japan), and swept-source OCT/OCTA (SS-OCT/OCTA) (VG200S, SVision Imaging, Henan, China). Eligible patients were divided into two groups according to the presence or absence of PS: the NPS group, which included highly myopic eyes without PS, and the PS group, which included highly myopic eyes with PS.

### SS-OCT/OCTA image acquisition and the diagnosis of posterior staphyloma

The SS-OCT/OCTA system used in this study was SVision Imaging from Henan, China (v.1.42.6). This system contained an swept-source laser with a center wavelength of approximately 1050 nm and a scan rate of 200,000 A-scans per second [14], which was also equipped with an eye-tracking device which eliminated eye movement artefacts. All patients underwent 36 radial scans of 16 mm length and 6 mm depth, centered on the fovea. The patient also underwent a 6 × 6 mm cube scan centered on the fovea. The macular area was divided into foveal and parafoveal regions. The foveal region was a circular area with a diameter of 1 mm, centered on the fovea. The parafoveal region was a ring area around the fovea with an inner diameter of 1 mm and an outer diameter of 3 mm (Figure S1). The scan size was adjusted based on the differences in magnification caused by the different ALs of the eyes, resulting in more accurate results. Bennett’s formula was used to determine the scaling factor of the OCT angiograms to adjust the ocular magnification in highly myopic eyes [scaling factor = 3.382 × 0.013062 × (AL − 1.82)] [15].

In our study, for a patient to be diagnosed with PS, the following characteristics had to be met: the thickness of the choroid was to be the thinnest at the edge of the staphyloma and thickened gradually from the edge to the periphery and the posterior pole of the fundus [13, 16]. The PS was judged to be present when the above mentioned characteristics were observed in a minimum of nine consecutive cross-sections in the 36 radial scans [16]. Since our study focused on the macula, the types of PS we included were mainly types I and II [13].

### Measurement of macular curvature and other parameters

**Fig. 1:**
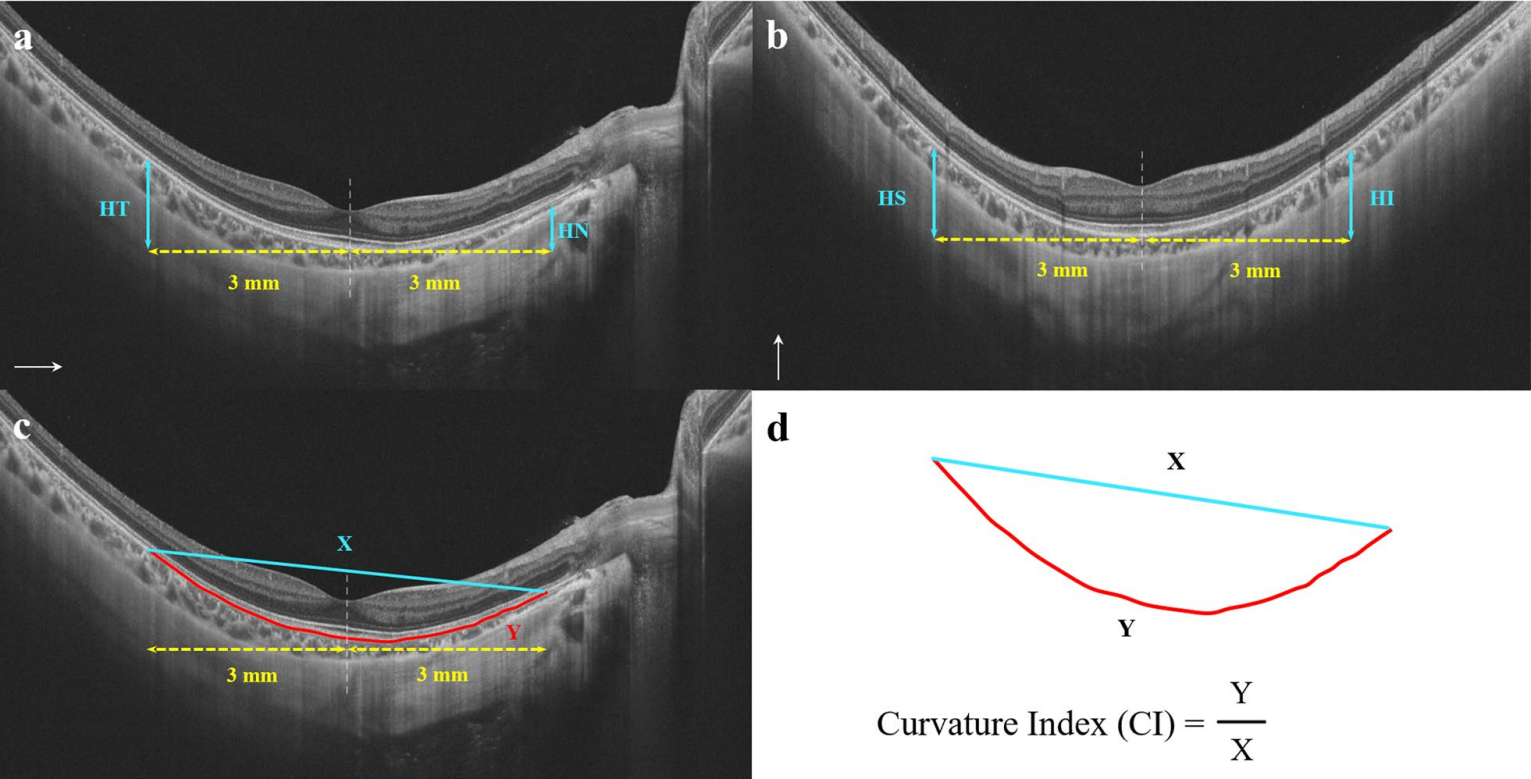
Measurement of PSH and CI. **a** and **b** PSH was defined as the summation of the vertical distance of the nasal, temporal, superior, and inferior positions from the fovea to 3 mm outside the fovea at the level of the retinal pigment epithelium (RPE). PSH = HT + HN + HS + HI. **c** and **d** CI was used to evaluate macular curvature. It is defined as the ratio of the length of the RPE line between two points 3 mm from the fovea to the straight line distance between the two points. CI = Y/X. PSH, posterior scleral height; CI, curvature index

Posterior scleral height (PSH) was defined as the summation of the vertical distance of the nasal, temporal, superior, and inferior positions from the fovea to 3 mm outside the fovea at the level of the retinal pigment epithelium (RPE), PSH = HT + HN + HS + HI (Fig. 1) [17, 18]. The curvature index (CI) was used to evaluate macular curvature, defined as the ratio of the length of the RPE line between two points 3 mm from the fovea to the straight line distance between the two points (Fig. 1) [19]. The automation results recorded include inner retinal vascular density (RVD), choriocapillaris perfusion area (CCPA), choroid perfusion area (CPA), choroidal vascularity index (CVI) and choroidal vessel volume (CVV) of the fovea and parafoveal region (Figure S2). The inner retina was automatically defined by the system as the area from 5 μm above the inner limiting membrane to 25 μm below the lower border of the inner nuclear layer. The choroid was defined as the area from the RPE-Bruch's membrane complex to the choroid-sclera interface. The choriocapillaris was defined as the microvasculature from the basal border of the RPE-Bruch's membrane complex to 20 μm below [20]. CVI was defined as the ratio of CVV to the total choroidal area. For calculating microcirculatory parameters, the blood flow signal intensity was used to differentiate between pixels indicating blood flow and those representing non-blood flow, by means of an empirical threshold. Subsequently, the total number of blood flow pixels was counted and converted into the final flow area, accounting for the imaging dimensions. Choroidal thickness (CT) was defined as the vertical distance between the outer edge of the RPE line and the outer edge of the choroid. Scleral thickness (ST) was defined as the vertical distance between the choroidoscleral interface and the outer edge of the sclera. Subfoveal choroidal thickness (SFCT) and subfoveal scleral thickness (SFST) were measured manually using a built-in caliber tool within the software (Figure S1). Choroidal and scleral thicknesses at the fovea were recorded as mean values measured in horizontal and vertical OCT scans.

### Statistical analysis

Statistical analyses were performed using SPSS software (v. 26.0, SPSS, IBM, Chicago) and R (v.4.0.2). Normality of data was tested using the Kolmogorov–Smirnov test or the Shapiro–Wilk test, as appropriate. The Chi-squared test, Fisher's exact test, independent t-test and Mann–Whitney U test were used when appropriate. BCVA was converted to the logarithm of the minimum resolution angle of resolution (logMAR) for statistical analysis.

Logistic regression for constructing PS diagnostic model using R (v.4.0.2). To eliminate the collinearity among the variables, the variables were first screened using machine learning methods. Samples were randomly divided into training and test sets at a ratio of 2:1. Support vector machine (SVM), random forest (RF), and least absolute shrinkage and selection operator (LASSO) models were trained on the training set. All models were cross-validated 10 times. Model performance was evaluated on the test set using receiver operating characteristic (ROC) curves. The variables selected by the best performing model were then subjected to univariate logistic regression and multivariate stepwise logistic regression for constructing a diagnostic model as well as a risk prediction model for the progression of PS. Single-factor logistic regression on all variables was performed, and select variables with *P* < 0.2 were included in multivariable stepwise logistic regression. A best fit model was created and variables were extracted from it to construct the final predictive model, and lastly, plot a nomogram. Fitted curves were plotted using locally weighted regression. All quantitative measurements were expressed as mean ± standard deviation (SD). A *P* value of less than 0.05 indicates a statistically significant difference.

## Results

### Baseline and mean changes per year in studied eyes

Characteristics of the studied eyes are shown in **Table S1**. Among the 122 eyes of 122 patients, 64 eyes with PS (PS group) and the other 58 eyes without PS (NPS group). The follow-up period was similar between the two groups, averaging 23.60 ± 9.15 months for the NPS group and 24.19 ± 8.49 months for the PS group. Patients in the PS group had significantly higher spherical equivalent refraction (SER) values (− 12.73 ± 3.52 D) compared to the NPS group (− 8.89 ± 2.00 D, *P* < 0.001). BCVA was worse in the PS group (0.12 ± 0.27 logMAR) compared to the NPS group (0.03 ± 0.09 logMAR, *P* = 0.001). AL was longer in the PS group (*P* < 0.001). SFCT and SFST were both significantly reduced in the PS group compared to the NPS group (all *P* < 0.001). Foveal and parafoveal CT, as well as CI, showed significant differences between two groups, with lower values in the PS group compared to the NPS group (all *P* < 0.001). PSH was significantly greater in the PS group (1378.16 ± 457.10 µm) than in the NPS group (784.09 ± 218.11 µm, *P* < 0.001). CCPA, CPA, and CVI were significantly lower in the PS group across both foveal and parafoveal regions compared to the NPS group (all *P* < 0.001).

The PS group experienced a greater mean annual increase in myopia (− 0.51 ± 0.31 D) compared to the NPS group (− 0.36 ± 0.29 D, *P* = 0.001). The PS group showed a slight worsening in BCVA (0.02 ± 0.05 logMAR) on average per year, compared to no change in the NPS group (0.00 ± 0.04 logMAR, *P* = 0.011). The mean annual increase in AL was greater in the PS group (0.15 ± 0.11 mm) than in the NPS group (0.09 ± 0.07 mm, *P* < 0.001). There was a significant difference in the mean change in CI between the groups, with the PS group showing less change (*P* < 0.001). The PS group exhibited a significantly greater increase in PSH (74.84 ± 75.48 µm) compared to the NPS group (40.26 ± 67.70 µm, *P* = 0.001). Significant differences were observed in the changes of CCPA, CPA and CVI between the groups, favoring the PS group (all *P* < 0.05).

### Risk factors and diagnostic model for PS

Dimensionality reduction within datasets exhibiting multicollinearity, facilitated through machine learning, and allows for the extraction of refined data that is subsequently integrated into linear regression analysis. The results of the univariate and multivariate stepwise logistic regression are shown in Table S2. The ROC curves of the three machine learning models showed that LASSO model had the largest area under the curve (AUC = 0.92), so the variables screened by this model were chosen as the final characteristic variables (Figure S3a). The results showed that baseline SER (B =  − 1.291, OR = 0.275, *P* = 0.008), baseline SFST (B =  − 1.621, OR = 0.198, *P* = 0.046), baseline PSH (B = 2.959, OR = 19.282, *P* = 0.001) and foveal CVI changes per year (B =  − 2.776, OR = 0.062, *P* < 0.001) were risk factors for PS. The diagnostic model (AUC = 0.97) was finally constructed using AL changes per year, foveal CT changes per year, baseline SER, baseline SFST, baseline PSH and foveal CVI changes per year (Figure S3b). The nomogram based on the diagnostic model is shown in Fig. 3.

### Correlation between AL and the mean change per year of other parameters

**Fig. 2:**
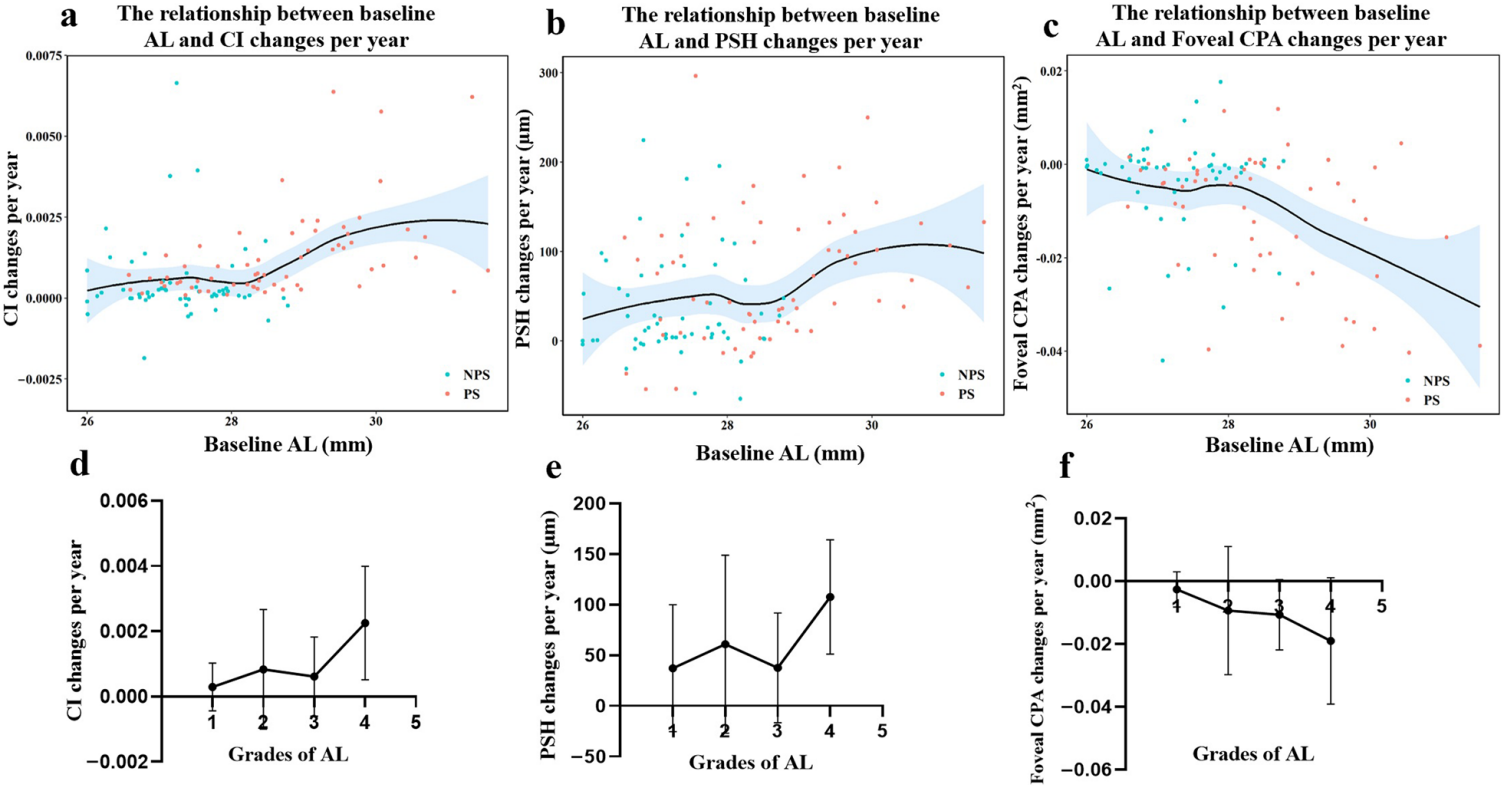
Fitted curves of baseline AL and CI changes per year (**a**), PSH changes per year (**b**) and foveal CPA changes per year (**c**). The red dots in the figure represent the sample distribution of the PS group and the blue dots represent the sample distribution of the NPS group. The variation of CI changes per year (**d**), PSH changes per year (**e**) and foveal CPA changes per year (**f**) for different baseline AL grades. Grade 1 was defined as: 26 mm ≤ AL < 27 mm; Grade 2: 27 mm ≤ AL < 28 mm; Grade 3: 28 mm ≤ AL < 29 mm; Grade 4: AL ≥ 29 mm. AL, axial length; CI, curvature index; PSH, posterior scleral height; PS, posterior staphyloma; NPS, without posterior staphyloma; CPA, choroidal perfusion area

**Fig. 3:**
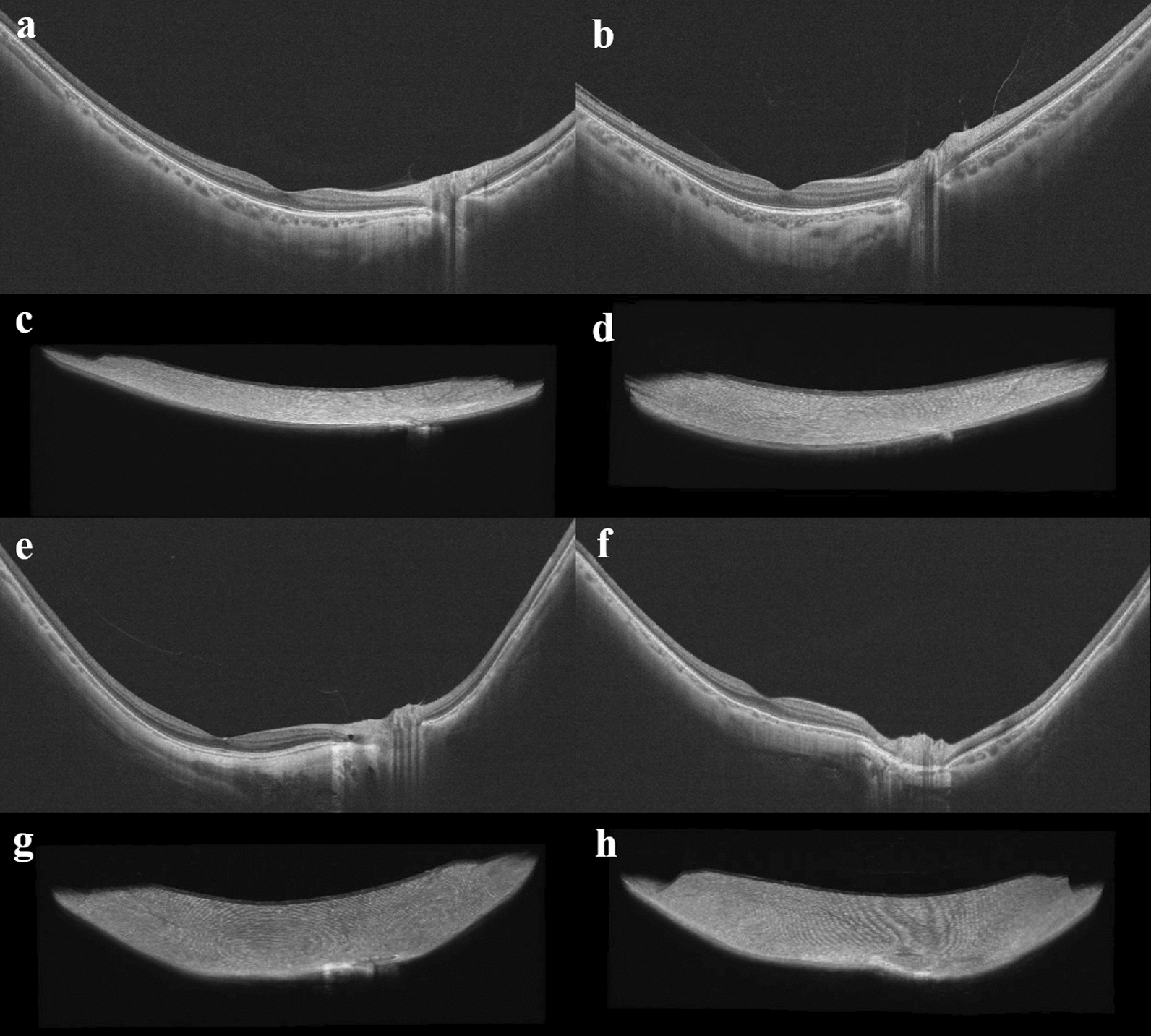
Optical coherence tomography (OCT) and three-dimensional (3D) images of highly myopic adult eyes. The 3D images display a perspective from above the eyeball. **a** and **c** are from a patient with an axial length (AL) of 26.60 mm. **b** and **d** are from a patient with an AL of 27.51 mm. **e** and **g** are from a patient with an AL of 28.96 mm. **f** and **h** are from a patient with an AL of 30.10 mm

The curves for CI changes per year, PSH changes per year, foveal CPA changes per year, and baseline AL were fitted separately using locally weighted regression (Fig. 2). To further clarify the change in macular morphology in myopia progression with different AL, we categorized baseline AL into four grades. Grade 1 was defined as: 26 mm ≤ AL < 27 mm; Grade 2: 27 mm ≤ AL < 28 mm; Grade 3: 28 mm ≤ AL < 29 mm; Grade 4: AL ≥ 29 mm. The variation of CI changes per year, PSH changes per year and foveal CPA changes per year for different baseline AL grades are shown in Fig. 2. Subsequently, we compared the morphology of the posterior pole in eyes with different AL (Fig. 3). Baseline AL was significantly correlated with CI changes per year (r = 0.335, *P* < 0.001), PSH changes per year (r = 0.250, *P* = 0.006), foveal CPA changes per year (r =  − 0.283, *P* = 0.001) and foveal CVI changes per year (r =  − 0.347, *P* < 0.001) (Figure S4).

### Baseline and mean changes per year in eyes with PS

Table 1 shows the differences between subgroups with PSH mean changes per year below or above the median level of 50 µm (designated as the slow progression and fast progression subgroups, respectively). Patients with PSH mean changes per year of ≥ 50 µm were defined as having progression of PS (i.e., aggravated PS subgroup). Those with PSH mean changes per year < 50 µm were named as the stabilized PS subgroup. PSH progression < 50 µm was observed in 31 patients. PSH progression ≥ 50 µm was observed in 33 patients. Baseline AL was significantly higher in the aggravated PS subgroup (28.30 ± 0.88 mm) than in the stabilized PS subgroup (28.94 ± 1.38 mm, *P* = 0.030). Foveal RVD changes per year and foveal CCPA changes per year were significantly higher in the aggravated PS subgroup than in the stabilized PS subgroup (all *P* < 0.05). No significant differences in other baseline characteristics were seen between the two groups (all *P* > 0.05).

**Table 1:** Baseline and mean changes per year in eyes with PS

Parameters	Progression < 50 µm (n = 31)	Progression ≥ 50 µm (n = 33)	*P* value
Baseline characteristics			
Age (years)	35.52 ± 12.74	40.30 ± 14.65	0.181
Sex (male/female)	11/20	9/24	0.479
AL (mm)	28.30 ± 0.88	28.94 ± 1.38	**0.030**
SER (D)	− 12.08 ± 3.50	− 13.33 ± 3.47	0.156
BCVA (logMAR)	0.08 ± 0.16	0.15 ± 0.34	0.831
SFCT (µm)	118.15 ± 60.88	97.22 ± 55.23	0.078
SFST (µm)	324.90 ± 62.66	311.40 ± 79.57	0.456
Foveal CT (µm)	147.88 ± 74.43	130.05 ± 63.99	0.295
Parafoveal CT (µm)	151.11 ± 66.59	133.13 ± 60.68	0.248
CI	1.018 ± 0.002	1.020 ± 0.004	0.072
PSH (µm)	1401.58 ± 468.15	1356.16 ± 452.60	0.695
Foveal RVD (%)	16.06 ± 6.62	17.33 ± 9.21	0.529
Parafoveal RVD (%)	63.43 ± 7.14	61.32 ± 12.61	0.809
Foveal CCPA (mm^2^)	0.61 ± 0.11	0.61 ± 0.13	0.946
Parafoveal CCPA (mm^2^)	4.82 ± 0.62	4.75 ± 0.99	0.658
Foveal CPA (mm^2^)	6.25 ± 0.06	6.18 ± 0.25	0.752
Parafoveal CPA (mm^2^)	0.78 ± 0.01	0.77 ± 0.04	0.502
Foveal CVI	0.34 ± 0.18	0.31 ± 0.17	0.476
Parafoveal CVI	0.38 ± 0.14	0.33 ± 0.16	0.119
Mean changes per year of the characteristics			
AL (mm)	0.12 ± 0.06	0.17 ± 0.14	0.113
SER (D)	− 0.45 ± 0.24	− 0.57 ± 0.36	0.164
BCVA (logMAR)	0.02 ± 0.05	0.02 ± 0.06	0.377
SFCT (µm)	− 1.69 ± 6.47	− 3.31 ± 14.21	0.168
SFST (µm)	− 2.69 ± 23.33	− 8.42 ± 12.76	0.199
Foveal CT (µm)	− 4.43 ± 6.18	− 6.11 ± 7.57	0.337
Parafoveal CT (µm)	− 4.91 ± 6.32	− 7.58 ± 7.62	0.336
CI	0.001 ± 0.001	0.002 ± 0.002	0.062
Foveal RVD (%)	− 2.35 ± 6.01	2.67 ± 9.55	**0.035**
Parafoveal RVD (%)	0.20 ± 4.87	3.54 ± 7.18	0.123
Foveal CCPA (mm^2^)	− 0.02 ± 0.04	− 0.04 ± 0.05	**0.023**
Parafoveal CCPA (mm^2^)	− 0.13 ± 0.32	− 0.16 ± 0.39	0.163
Foveal CPA (mm^2^)	− 0.02 ± 0.06	− 0.04 ± 0.08	0.168
Parafoveal CPA (mm^2^)	− 0.01 ± 0.01	− 0.02 ± 0.02	0.120
Foveal CVI	− 0.03 ± 0.02	− 0.04 ± 0.04	0.584
Parafoveal CVI	0.00 ± 0.03	0.00 ± 0.04	0.759

*P* values in bold indicate statistical significance

*AL* = axial length; *SER* = spherical equivalent refraction; *BCVA* = best-corrected visual acuity; *logMAR* = logarithm of the minimum resolution angle of resolution; *SFCT* = subfoveal choroid thickness; *SFST* = subfoveal scleral thickness; *CT* = choroid thickness; *CI* = curvature index; *PSH* = posterior scleral height; *RVD* = retinal vessel density; *CCPA* = choriocapillaris perfusion area; *CPA* = choroidal perfusion area; *CVI* = choroidal vascularity index

### Risk factors and prediction model for the PS progression

**Fig. 4:**
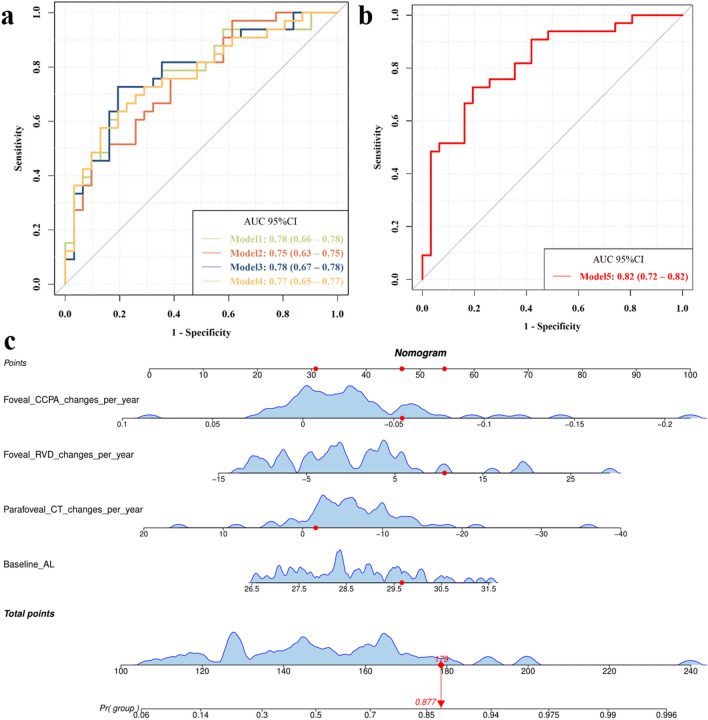
Predictive model and nomogram of posterior staphyloma (PS) progression. **a** Receiver operating characteristic (ROC) of the prediction models for PS progression. Model 1: baseline AL + parafoveal CT changes per year + foveal RVD changes per year. Model 2: baseline AL + parafoveal CT changes per year + foveal CCPA changes per year. Model 3: foveal RVD changes per year + parafoveal CT changes per year + foveal CCPA changes per year. Model 4: foveal RVD changes per year + baseline AL + foveal CCPA changes per year. **b** PS progression risk prediction model ROC curve with an area under the curve (AUC) of 0.820. Model 5: foveal CCPA changes per year + foveal RVD changes per year + parafoveal CT changes per year + baseline AL. **c** Nomogram for predicting the probability of PS progression. Patient 2 from this study is used as an example (presented in red). The foveal CCPA changes per year was − 0.055 mm^2^, foveal RVD changes per year was 10.64%, parafoveal CT changes per year was − 1.65 µm and baseline AL was 29.66 mm. Red lines and dots are drawn upward to determine the points received by each variable; the sum (179) of these points is located on the Total Points axis, and a line is drawn downward to the axes to determine probability of PS progression (87.7%). AL, axial length; CT, choroid thickness; RVD, retinal vascular density; CCPA, choriocapillaris perfusion area

The results of univariate logistic regression and multifactor stepwise logistic regression with PS progression as the dependent variable were shown in Table 2. Variables with *P* < 0.2 from the results of the univariate logistic regression were included in the multivariate stepwise logistic regression. Baseline AL (B = 0.561, OR = 1.752, *P* = 0.033), parafoveal CT changes per year (B =  − 0.094, OR = 0.910, *P* = 0.032), foveal RVD changes per year (B = 0.104, OR = 1.110, *P* = 0.013) and foveal CCPA changes per year (B =  − 0.214, OR = 0.807, *P* = 0.038) were risk factors for the PS progression. They were subsequently used to construct risk prediction models. The ROC curves and nomogram of the prediction model for the PS progression are shown in Fig. 4.

**Table 2:** Univariate logistic regression and multivariate stepwise logistic regression with PS progression as the dependent variable

Characteristics	Univariate				Multivariate			
	B	OR	95% CI	*P*	B	OR	95% CI	*P*
Age	0.026	1.026	0.990–1.066	0.168				
Baseline AL	0.481	1.618	1.050–2.622	0.037	0.561	1.752	1.072–3.046	**0.033**
Baseline SER	− 0.106	0.900	0.772–1.038	0.156				
Baseline BCVA	1.059	2.883	0.397–54.961	0.360				
Baseline SFCT	− 0.006	0.994	0.985–1.002	0.155				
Baseline SFST	− 0.003	0.997	0.990–1.004	0.450				
Baseline foveal CT	− 0.004	0.996	0.989–1.003	0.304				
Baseline parafoveal CT	− 0.005	0.995	0.987–1.003	0.260				
Baseline CI	1.580	4.854	1.017–31.385	0.065				
Baseline PSH	0.000	1.000	0.999–1.001	0.689				
Baseline foveal RVD	0.020	1.021	0.959–1.089	0.523				
Baseline parafoveal RVD	− 0.021	0.979	0.928–1.028	0.414				
Baseline foveal CCPA	− 0.043	0.958	0.015–60.863	0.984				
Baseline parafoveal CCPA	− 0.107	0.899	0.479–1.643	0.727				
Baseline foveal CPA	− 3.100	0.045	0.000–1.512	0.175				
Baseline parafoveal CPA	− 12.182	0.000	0.000–99.845	0.213				
Baseline foveal CVI	− 1.026	0.358	0.018–6.407	0.488				
Baseline parafoveal CVI	− 2.249	0.105	0.003–2.822	0.190				
AL changes per year	0.055	1.057	1.000–1.137	0.091				
BCVA changes per year	0.011	1.011	0.914–1.129	0.827				
SER changes per year	− 1.446	0.236	0.031–1.306	0.124				
SFCT changes per year	− 0.014	0.987	0.938–1.032	0.560				
SFST changes per year	− 0.019	0.981	0.946–1.01	0.244				
Foveal CT changes per year	− 0.036	0.964	0.892–1.036	0.334				
Parafoveal CT changes per year	− 0.060	0.942	0.862–1.015	0.144	− 0.094	0.910	0.826–0.986	**0.032**
CI changes per year	0.578	1.782	1.110–3.387	0.041				
Foveal RVD changes per year	0.086	1.089	1.018–1.182	0.023	0.104	1.110	1.030–1.218	**0.013**
Parafoveal RVD changes per year	0.097	1.102	1.010–1.224	0.045				
Foveal CCPA changes per year	− 0.161	0.852	0.712–0.979	0.045	− 0.214	0.807	0.643–0.967	**0.038**
Parafoveal CCPA changes per year	− 0.003	0.997	0.983–1.012	0.722				
Foveal CPA changes per year	− 0.024	0.977	0.898–1.047	0.518				
Parafoveal CPA changes per year	− 0.035	0.965	0.922–1.001	0.090				
Foveal CVI changes per year	− 0.012	0.988	0.965–1.005	0.234				
Parafoveal CVI changes per year	− 0.002	0.998	0.982–1.013	0.757				

Variables with *P* < 0.2 in the univariate logistic regression were included in the multivariate stepwise logistic regression. *P* values in bold indicate statistical significance

*OR* = odds ratio; 95% *CI* = 95% confidence interval; *AL* = axial length; *SER* = spherical equivalent refraction; *BCVA* = best-corrected visual acuity; *SFCT* = subfoveal choroid thickness; *SFST* = subfoveal scleral thickness; *CT* = choroid thickness; *CI* = curvature index; *PSH* = posterior scleral height; *RVD* = retinal vessel density; *CCPA* = choriocapillaris perfusion area; *CPA* = choroidal perfusion area; *CVI* = choroidal vascularity index

## Discussion

In recent years, the morphology of the eyeball has been mainly assessed using three-dimensional magnetic resonance imaging (3D-MRI) [5]. However, this method only provides a qualitative evaluation for highly myopic eyes. There is no standardized method to quantitatively assess the height of PS and macular curvature. This study presents new longitudinal data investigating changes in PS height and macular curvature using the two methods mentioned above [18, 19]. To our knowledge, this study is the first to report on changes in macular morphology and microcirculation over time in highly myopic eyes.

We found significant differences in macular microstructure and microcirculation between highly myopic eyes with PS and without PS. This result was consistent with our previous findings [11]. Abnormalities in macular microcirculation have been confirmed in several studies as a risk factor for high myopia and its complications [7–10]. Although some studies have not found significant differences in macular CVI between healthy myopic and myopic traction maculopathy patients [21], abnormalities in microcirculation around the optic disc can be observed in myopic traction maculopathy [22]. The reduction in blood flow density in the retina was significantly higher in highly myopic eyes than in non-highly myopic eyes and was strongly associated with a larger baseline AL [23, 24]. However, our study found no significant difference in the mean annual loss of RVD between eyes with and without PS. This suggests that changes in the RVD are more pronounced before pathologic changes occur in highly myopic eyes, but after the onset of complications such as PS, the decrease in choroidal blood flow becomes the dominant factor. During the longitudinal follow-up, the PS group showed a faster rate of AL growth and a faster rate of decrease with regards to the CT, CCPA, CPA and CVI vs. the NPS group. Generally, AL growth rate stabilizes after 20 years of age [25], but the phenomenon of AL growth in myopic patients seems to progress over an extended period. The mean AL growth rate in the stable progression trajectory was 0.12 mm/year [26], compared with a faster AL progression rate in highly myopic eyes with PS in our study (mean growth rate of 0.15 mm/year). In early adulthood, CVI increased at a rate of 0.07 per year, and luminal area increased at a rate of 0.01 per year [27]. Over time, myopic macular degeneration worsens in 1 out of every 10 eyes in adults [28]. Similarly, we found that foveal CVI decreased by an average of 0.01 per year in the adult high myopia population, and in eyes with PS, the decrease in CVI was even more significant, up to an average decrease of 0.03 per year. Macular curvature and PSH in eyes with PS steepened more dramatically during myopic progression, meaning that the decrease in scleral biomechanics is more significant in eyes with PS. The results showed that baseline SER, baseline SFST, baseline PSH and foveal CVI changes per year were risk factors for PS. As reported by other researchers [29–31], the decrease in choroidal blood flow leads to insufficient oxygen and nutrient supply to the sclera. This results in a phenotypic shift from scleral fibroblasts to myofibroblasts and a decrease in collagen production. Consequently, the sclera becomes thinner and more fragile. The thinned sclera has less cushioning against intraocular pressure as well as less resistance to AL elongation, resulting in the development of PS. In addition, the heterogeneity in the curvature of the choroid and sclera in PS eyes may also lead to tissue remodeling in the corresponding areas, resulting in changes in the microstructure and microvasculature. This may lead to abnormalities in blood circulation, which may further compromise retinal function. Long-term longitudinal nonbiased studies with blinded examiners are needed to determine these findings more reliably.

**Fig. 5:**
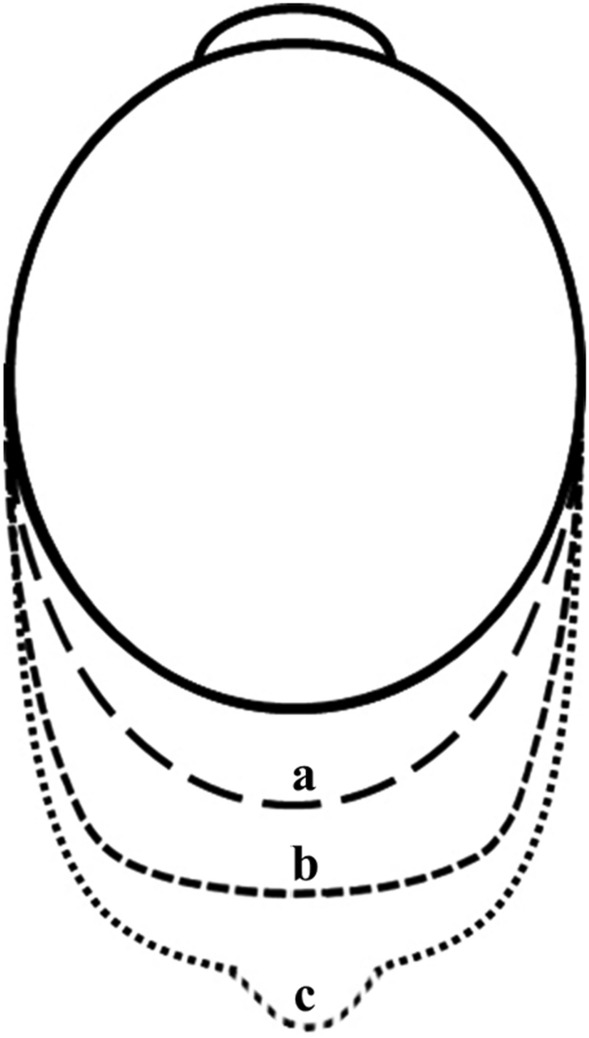
An evolutionary pattern of posterior staphyloma (PS). The evolution of highly myopic eyes over time is displayed by the dotted line. Dotted line (**a**) represents the longitudinal elongation of the eye; dotted line (**b**) indicates the transversal elongation of the posterior pole of the fundus; dotted line (**c**) shows the appearance of PS

To explore the progression of PS at different stages, we examined the trends in the different baseline AL grades of CI changes per year, PSH changes per year, and foveal CPA changes per year. Based on these results, we observed the morphological features of the posterior pole of the fundus in patients with different AL. We preliminarily discovered the fact that the posterior pole of the fundus expanded longitudinally, then transversely, and finally appeared as a localized bulge of the eyeball (Fig. 5). As myopia progresses, the CI and PSH gradually increase, causing longitudinal expansion of the eyeball. Subsequently, the CI stabilizes while the PSH decreases slightly, and the posterior pole of the fundus expands transversely, indicating a possible compensation for the changes in morphology. However, if the biomechanical compensatory limits of the posterior pole of the eyeball are exceeded, it may lead to localized posterior bulging of the eyeball, with a significant increase in the CI and PSH, and PS may appear. This process is accompanied by changes in macular microcirculation that are synchronized and closely related to myopic progression. The rate of decrease in macular blood flow can be used to predict the risk of PS progression. This is a new perspective to characterize the evolutionary pattern of PS, although the process of PS evolution may be diverse, the ultrastructural mechanism behind this process still deserves further exploration.

The choroid was involved in transmitting visual signals from the retina to the sclera, mediating the progression of myopia [32]. It has been shown that early changes in choroidal vasculature can serve as a predictive biomarker for long-term myopia control effects [33]. Microcirculatory abnormalities in the retina were similarly observed in eyes with PS [34]. In adults, CVI can be used as a biomarker to predict the transition to myopia as well as visual function in patients with high myopia [35, 36]. These highlight the potential role of changes in the fundus microcirculation in predicting myopia progression. In this study, baseline AL, parafoveal CT changes per year, foveal RVD changes per year and foveal CCPA changes per year were risk factors for PS progression in highly myopic adult eyes. In this case, the probability of PS progression can be predicted using the nomogram (Fig. 4c). Furthermore, this study found that the larger the baseline AL, the faster the CI and PSH increased and the faster the CPA and CVI were lost. This suggests that a larger baseline AL increases the chances of PS onset and progression.

When patients are predicted to be at high risk of PS progression, clinicians can promptly intervene with enhanced interventions or even perform surgery such as posterior scleral reinforcement surgery to prevent further aggravation of PS as well as the development of other myopic complications.

### Study strengths and limitations

The main strengths of this study are that it is a longitudinal study and it is the first to investigate the relationship between changes in the microcirculation and the progression of PS. Moreover, this study is the first to propose a possible evolutionary pattern of PS, providing new insights into the pathogenesis of pathologic myopia.

The limitations of this study should also be noted. The small sample size did not allow us to completely exclude the presence of retinal schisis and the effect of different levels of chorioretinal atrophy, which will be improved by increasing the number of follow-up patients in the future. The study lacked the inclusion of children since there is a low incidence of PS in children with highly myopic eyes [37]. The pattern of PS evolution in children may also be different from that in adults. Taken together, a separate study studying PS in children may be warranted.

## Conclusion

Changes in choroidal microcirculation can be the risk factor for diagnosing the presence of PS and for predicting PS progression. Our preliminary findings suggest that there perhaps exist a compensatory stage in the evolution of PS that enables the posterior pole of the eye to expand laterally toward the periphery of the macula to resist excessive elongation of the AL. These findings demonstrate a significant role of microcirculatory changes in the progression of PS for the first time and provided new insights for predicting the deterioration of PS in the future and for timely intervention.

## Supplementary Information

Supplementary Material 1.

## Data Availability

The data and materials collected in this study are available upon request from the corresponding authors.

## Abbreviations

AL: Axial length
BCVA: Best-corrected visual acuity
CCPA: Choriocapillaris perfusion area
CI: Curvature index
CPA: Choroid perfusion area
CT: Choroidal thickness
CVI: Choroidal vascularity index
CVV: Choroidal vessel volume
IOP: Intraocular pressure
LASSO: Least absolute shrinkage and selection operator
OCT: Optical coherence tomography
OCTA: Optical coherence tomography angiography
PS: Posterior staphyloma
PSH: Posterior scleral height
RF: Random forest
RPE: Retinal pigment epithelium
RVD: Retinal vascular density
SER: Spherical equivalent refraction
SFCT: Subfoveal choroidal thickness
SFST: Subfoveal scleral thickness
SS-OCT/OCTA: Swept-source OCT/OCTA
ST: Scleral thickness
SVM: Support vector machine
3D-MRI: Three-dimensional magnetic resonance imaging
