# Supplementary material for: Microcirculatory parameters as risk factors for predicting progression of posterior staphyloma in highly myopic eyes: a case–control study
Source: Eye Vis (Lond). 2024 Dec 1;11:45. doi: 10.1186/s40662-024-00413-1 (PMC11608478; doi:10.1186/s40662-024-00413-1)
Supplement: Supplementary file 1 — Supplementary Material 1. [file 40662_2024_413_MOESM1_ESM.docx]

**Supplemental material**

**Figure S1.** Two regions of macular and manually measured parameters. **a** The macular area was divided into foveal and parafoveal regions. The foveal region was a circular area with a diameter of 1 mm centered on the fovea. The parafoveal region was a ring area around the fovea with an inner diameter of 1 mm and an outer diameter of 3 mm (the grey ring area). **b** SFCT (red double arrow) and SFST (blue double arrow) were measured manually using a built-in caliber tool within the software. SFCT, subfoveal choroid thickness; SFST, subfoveal scleral thickness.


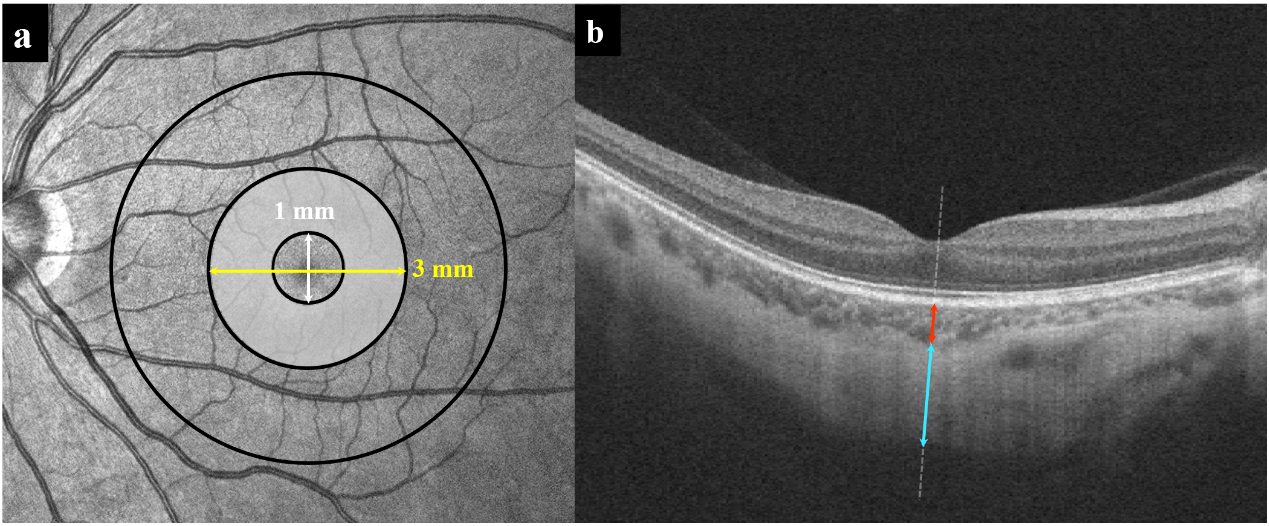


**Figure S2.** The value of microcirculation parameters is automatically calculated by the system. **a** Inner retinal vessel density (RVD); **b** Choroidal perfusion area (CPA); **c** Choroidal vascularity index (CVI). The top *en face* images are intensity projections of the regions marked by the blue lines in the bottom images.


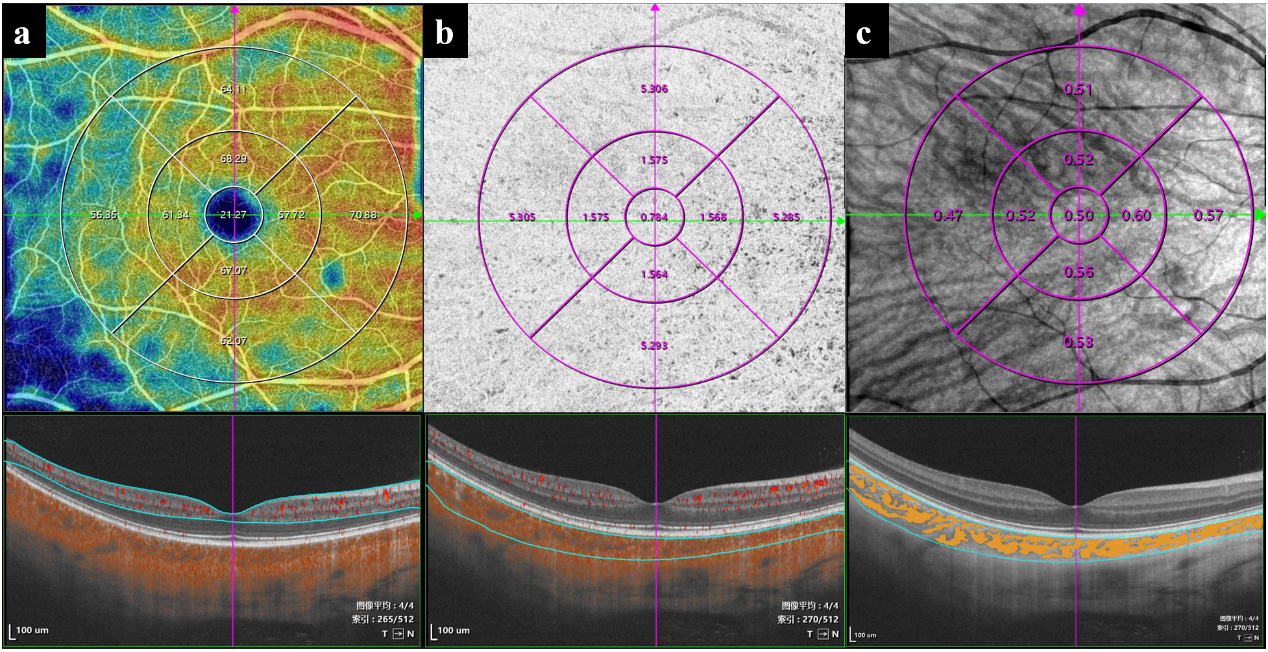


**Figure S3.** Diagnostic model and nomogram of posterior staphyloma (PS). **a** Model performance evaluation. Receiver operating characteristic (ROC) curves for the three models, yellow for the support vector machine (SVM) model, red for the random forest (RF) model, and green for the least absolute shrinkage and selection operator (LASSO) model. **b** PS diagnostic model ROC curve with an area under the curve (AUC) of 0.97. **c** Nomogram for diagnosing the probability of PS. Patient 1 from this study is shown as an example (presented in red). The foveal choroidal vascularity index (CVI) changes per year was −0.002, foveal choroid thickness (CT) changes per year was −1.33 µm, axial length (AL) changes per year was 0.03 mm, baseline posterior scleral height (PSH) was 970.8 µm, baseline subfoveal scleral thickness (SFST) was 389.8µm and baseline spherical equivalent refraction (SER) was −8.75 D. Red lines and dots are drawn upward to determine the points received by each variable; the sum (137) of these points is located on the Total Points axis, and a line is drawn downward to the axes to determine the probability of diagnosing PS (3.71%).


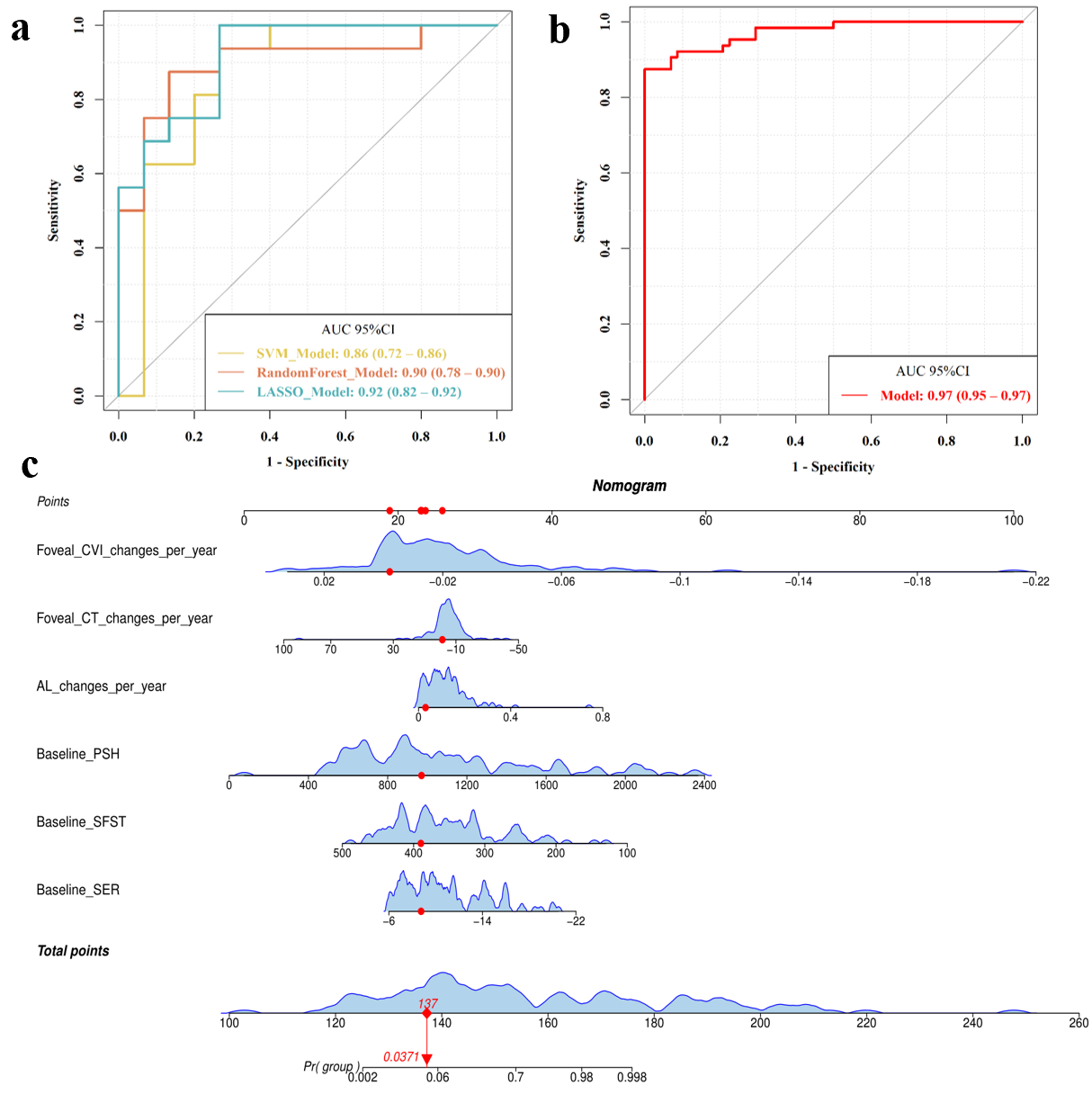


**Figure S4.** Baseline axial length (AL) was correlated with curvature index (CI) changes per year (**a**), posterior scleral height (PSH) changes per year (**b**), foveal choroidal perfusion area (CPA) changes per year (**c**) and foveal choroidal vascularity index (CVI) changes per year (**d**).


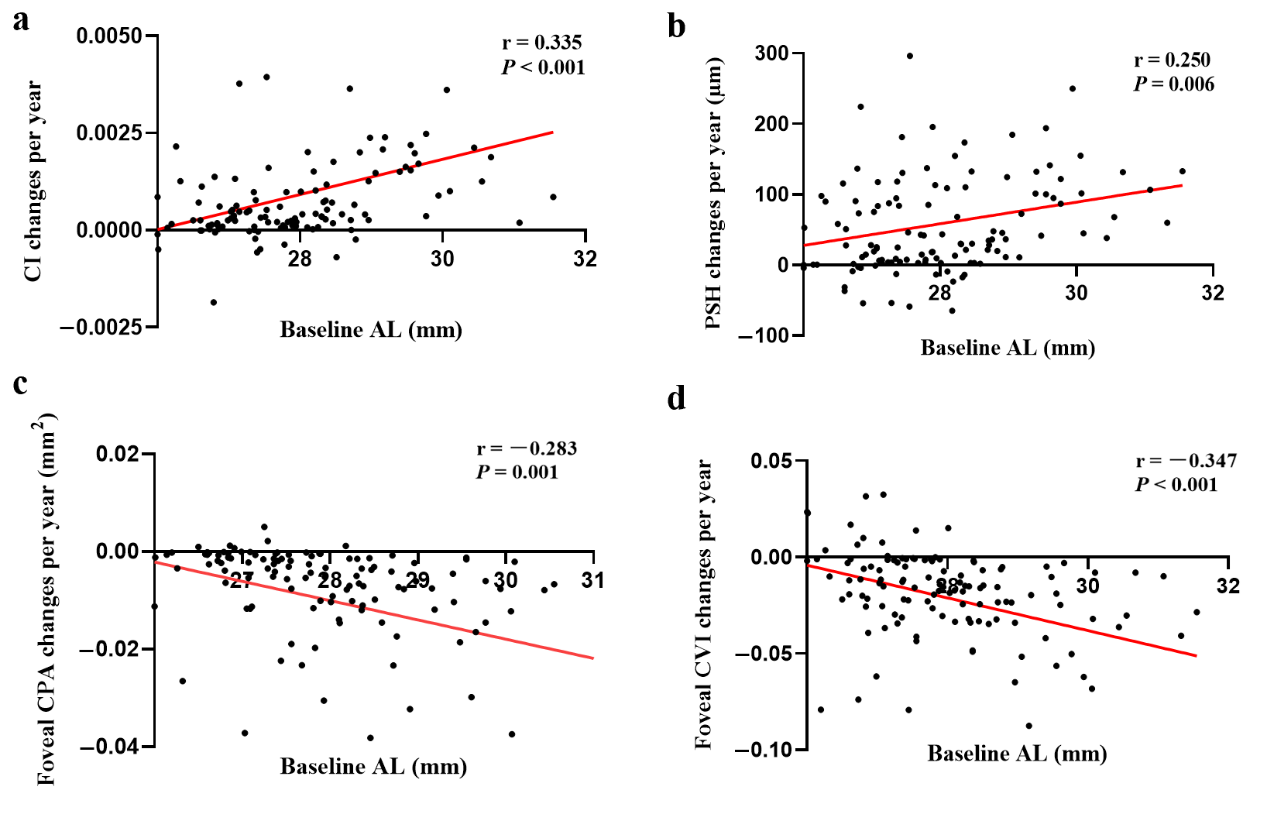


| **Table S1.** Baseline and mean changes per year of the studied eyes. | | | |
| --- | --- | --- | --- |
| **Parameters** | **NPS group**  **(n=58)** | **PS group**  **(n=64)** | ***P* value** |
| **Baseline characteristics of the studied eyes** | | | |
| Follow-up period (month) | 23.60 ± 9.15 | 24.19 ± 8.49 | 0.474 |
| Age (years) | 33.19 ± 10.67 | 37.98 ± 13.86 | 0.070 |
| Sex (male/female) | 21/37 | 20/44 | 0.572 |
| SER (D) | −8.89 ± 2.00 | −12.73 ± 3.52 | **< 0.001** |
| BCVA (logMAR) | 0.03 ± 0.09 | 0.12 ± 0.27 | **0.001** |
| AL (mm) | 27.31 ± 0.72 | 28.63 ± 1.20 | **< 0.001** |
| SFCT (µm) | 190.38 ± 39.1 | 107.36 ± 58.53 | **< 0.001** |
| SFST (µm) | 394.11 ± 45.42 | 317.94 ± 71.64 | **< 0.001** |
| Foveal CT (µm) | 226.12 ± 48.92 | 138.69 ± 69.27 | **< 0.001** |
| Parafoveal CT (µm) | 236.06 ± 50.43 | 141.84 ± 63.75 | **< 0.001** |
| CI | 1.014 ± 0.005 | 1.019 ± 0.003 | **< 0.001** |
| PSH (µm) | 784.09 ± 218.11 | 1378.16 ± 457.10 | **< 0.001** |
| Foveal RVD (%) | 18.99 ± 8.29 | 16.72 ± 8.02 | 0.238 |
| Parafoveal RVD (%) | 66.42 ± 6.89 | 62.34 ± 10.3 | **0.027** |
| Foveal CCPA (mm^2^) | 0.76 ± 0.69 | 0.61 ± 0.12 | **0.001** |
| Parafoveal CCPA (mm^2^) | 5.31 ± 0.88 | 4.79 ± 0.83 | **0.001** |
| Foveal CPA (mm^2^) | 0.79 ± 0.02 | 0.77 ± 0.03 | **< 0.001** |
| Parafoveal CPA (mm^2^) | 6.37 ± 0.68 | 6.22 ± 0.19 | **0.001** |
| Foveal CVI | 0.47 ± 0.08 | 0.32 ± 0.17 | **< 0.001** |
| Parafoveal CVI | 0.45 ± 0.07 | 0.36 ± 0.15 | **< 0.001** |
| **Mean changes per year of the characteristics** | | | |
| SER | −0.36 ± 0.29 | −0.51 ± 0.31 | **0.001** |
| BCVA | 0.00 ± 0.04 | 0.02 ± 0.05 | **0.011** |
| AL | 0.09 ± 0.07 | 0.15 ± 0.11 | **< 0.001** |
| SFCT | −8.08 ± 16.99 | −2.52 ± 11.1 | 0.068 |
| SFST | −9.08 ± 13.30 | −5.64 ± 18.72 | 0.156 |
| Foveal CT | −2.21 ± 16.54 | −5.3 ± 6.93 | 0.070 |
| Parafoveal CT | −7.88 ± 29.35 | −6.29 ± 7.09 | 0.113 |
| CI | 0.001 ± 0.002 | 0.001 ± 0.001 | **< 0.001** |
| PSH | 40.26 ± 67.70 | 74.84 ± 75.48 | **0.001** |
| Foveal RVD | −2.69 ± 4.75 | 0.24 ± 8.36 | 0.076 |
| Parafoveal RVD | −0.67 ± 6.90 | 1.92 ± 6.35 | 0.078 |
| Foveal CCPA | −0.05 ± 0.41 | −0.03 ± 0.04 | **0.009** |
| Parafoveal CCPA | 0.00 ± 0.57 | −0.15 ± 0.35 | **0.004** |
| Foveal CPA | −0.01 ± 0.02 | −0.01 ± 0.02 | **< 0.001** |
| Parafoveal CPA | −0.04 ± 0.42 | −0.03 ± 0.07 | **< 0.001** |
| Foveal CVI | −0.01 ± 0.02 | −0.03 ± 0.03 | **< 0.001** |
| Parafoveal CVI | 0.01 ± 0.05 | 0.00 ± 0.03 | 0.659 |

NPS = non-posterior staphyloma; PS = posterior staphyloma; SER = spherical equivalent refraction; BCVA = best-corrected visual acuity; logMAR = logarithm of the minimum resolution angle of resolution; AL = axial length; SFCT = subfoveal choroid thickness; SFST = subfoveal scleral thickness; CT = choroid thickness; CI = curvature index; PSH = posterior scleral height; RVD = inner retinal vessel density; CCPA = choriocapillaris perfusion area; CPA = choroidal perfusion area; CVI = choroidal vascularity index.

Variables with *P* < 0.2 in the univariate logistic regression were included in the multivariate stepwise logistic regression. *P* values in bold indicate statistical significance.

**Table S2.** Univariate logistic regression and multivariate stepwise logistic regression with posterior staphyloma (PS) as the dependent variable.

| **Characteristics** | **Univariate** | | | |  | **Multivariate** | | | |
| --- | --- | --- | --- | --- | --- | --- | --- | --- | --- |
|  | **B** | **OR** | **95% CI** | ***P*** |  | **B** | **OR** | **95% CI** | ***P*** |
| Baseline AL | 1.737 | 5.683 | 3.110–11.726 | < 0.001 |  |  |  |  |  |
| Baseline SER | −1.670 | 0.188 | 0.094–0.335 | < 0.001 |  | −1.291 | 0.275 | 0.093–0.671 | **0.008** |
| Baseline SFST | −1.634 | 0.195 | 0.097–0.349 | < 0.001 |  | −1.621 | 0.198 | 0.032–0.833 | **0.046** |
| Baseline PSH | 2.705 | 14.960 | 6.145–46.058 | < 0.001 |  | 2.959 | 19.282 | 4.328–155.014 | **0.001** |
| Baseline parafoveal CPA | −1.128 | 0.324 | 0.123–0.637 | 0.007 |  |  |  |  |  |
| AL changes per year | 0.918 | 2.505 | 1.506–4.491 | 0.001 |  | 0.717 | 2.049 | 0.900–5.373 | 0.113 |
| SFST changes per year | 0.227 | 1.254 | 0.866–1.931 | 0.258 |  |  |  |  |  |
| Foveal CT changes per year | −0.284 | 0.752 | 0.461–1.107 | 0.196 |  | −0.623 | 0.536 | 0.174–1.090 | 0.196 |
| PSH changes per year | 0.525 | 1.690 | 1.144–2.619 | 0.012 |  |  |  |  |  |
| Parafoveal CCPA changes per year | −0.373 | 0.689 | 0.417–1.029 | 0.103 |  |  |  |  |  |
| Foveal CVI changes per year | −1.715 | 0.180 | 0.077–0.365 | < 0.001 |  | −2.776 | 0.062 | 0.012–0.216 | < **0.001** |

OR = odds ratio; 95% CI = 95% confidence interval; AL = axial length; SER = spherical equivalent refraction; SFST = subfoveal scleral thickness; PSH = posterior scleral height; CPA = choroidal perfusion area; CT = choroid thickness; CCPA = choriocapillaris perfusion area; CVI = choroidal vascularity index.

*P* values in bold indicate statistical significance.
